# Physicochemical and molecular characterization of heavy metal–tolerant bacteria isolated from soil of mining sites in Nigeria

**DOI:** 10.1186/s43141-021-00251-x

**Published:** 2021-10-11

**Authors:** U. B. Ibrahim, A. H. Kawo, I. Yusuf, S. Yahaya

**Affiliations:** 1 Department of Microbiology, Faculty of Science, Usmanu Danfodiyo University, PMB 2346, Sokoto, Nigeria; 2 Department of Microbiology, Faculty of Life Sciences, College of Natural and Pharmaceutical Sciences, Bayero University Kano, PMB 3011, Kano, Nigeria

**Keywords:** Bacteria, Heavy metals, Tolerance, Soil, Mining

## Abstract

**Background:**

Mining for precious metals is detrimental to the composition of soil structure and microbial diversity distribution and is a health risk to human communities around the affected communities. This study was aimed at determining the physical and chemical characteristics and diversity of bacteria in the soil of local mining sites for biosorption of heavy metals.

**Results:**

Results of physical and chemical characteristics showed mean pH values and percentage organic carbon to range from 7.1 to 8.2 and 0.18 to 1.12% respectively with statistical significance between sampling sites (*P* ≤ 0.05). Similarly, cation exchange capacity, electrical conductivity, moisture, total nitrogen, and carbon/nitrogen ratio (C:N) in the soil ranged between 1.52 to 3.57 cmol/kg, 0.15 to 0.32 ds/m, 0.14 to 0.82%, 0.10 to 0.28%, and 1.7 to 4.8 respectively. The highest heavy metal concentration of 59.01 ppm was recorded in soils obtained from site 3. The enumeration of viable aerobic bacteria recorded the highest mean count of 4.5 × 10^6^ cfu/g observed at site 2 with statistical significance (*P* ≤ 0.05) between the sampled soils. *Alcaligenes faecalis* strain UBI, *Aeromonas* sp. strain UBI, *Aeromonas sobria*, and *Leptothrix ginsengisoli* that make up 11.2% of total identified bacteria were able to grow in higher amended concentrations of heavy metals. The evolutionary relationship showed the four heavy metal–tolerant bacteria identified belonged to the phylum Proteobacteria of class Betaproteobacteria in the order Burkholderiales. Heavy metal biosorption by the bacteria showed *Alcaligenes faecalis* strain UBI having the highest uptake capacity of 73.5% for Cu.

**Conclusion:**

In conclusion, *Alcaligenes faecalis* strain UBI (MT107249) and *Aeromonas* sp. strain UBI (MT126242) identified in this study showed promising capability to withstand heavy metals and are good candidates in genetic modification for bioremediation.

**Supplementary Information:**

The online version contains supplementary material available at 10.1186/s43141-021-00251-x.

## Background

The use of simple instruments and manual labor to extract precious metals is widespread nowadays. This activity has increased the risk of degradation of soil biota and the displacement of microorganisms and microbial structure, likewise destruction of soil texture and arrangement [1]. The adverse effect of these processes has led to serious opposition against this activity of mining [2]. Several authors have reported the serious impact mining has on the environment, especially by the number of waste materials produced that are non-beneficial to the surrounding environment [3]. The release of these mining products was potentially threatening to the huge population of people inhabiting these risk-averse areas [3]. Pandev and Kumar [4] itemized and put forward some effects of mining to include soil pollution, degradation, and clearing of vegetation and soil organic matter, and can eventually reduce biological activity and decrease soil productivity. This can lead to the soil environment being disrupted by modifying the physical and chemical contents and processes that might in one way or the other affect both living and non-living subject hosts in the affected environment [4]. Furthermore, pollutants from external sources such as industries, agricultural activities, traffic, etc. tend to influence the accumulation of such heavy metals and other wastes thereby hurting the immediate environment [5]. Although physical, biological, and chemical processes promote the movement of these chemicals across the soil horizon [1], the effect these movements have on the food chain was due to the passive nature of the soil, which hinder smooth interaction between soil humus and other fertility molecules [6]. Adewole and Adesina [7] posited that mining activities and mine waste generation, in addition to enriching soils with heavy metal (HM), could also affect nutrient dynamics in soils because of dynamic and interaction changes in physical, chemical, and microbiological processes. Several studies [8, 9] have highlighted the importance of soil parameters such as organic matter (OM), particle size distribution, clay content, redox potential, electrical conductivity (EC), moisture content, cation exchange capacity (CEC), and pH on heavy metal behavior in soils. Fashola et al. [9] maintained that metal mobility was found to be lower in fine-textured soils than in coarse-textured soils, particularly when the clayey soil’s mineralogical composition is dominated by 2:1 tetrahedral: octahedral silicate clay minerals. Acidic circumstances lower soil exchange capabilities of metal cations and increase metal solubility in the soil environment, making them more mobile, but a high level of organic matter can enhance metal adsorption, reducing mobility in the environment as emphasized by Ayangbenro et al. [10]. In another study, Fashola et al. [11] reported that acid mine drainage from abandoned goldmines has acidified surrounding soils, affecting HM mobility and microbiological diversity in the soil. For every unit decrease in soil pH, zinc solubility has been shown to rise 100-fold. Further studies by Ndeddy-Aka and Babalola [12] reported that in contaminated soils, increased solubility of Pb, Cd, and Zn was recorded as the pH declined from 5.0 to 3.3. Changes in soil pH disrupt specific microbial metabolic processes by blocking pH-dependent enzymes’ activity or affecting the availability of essential nutrients and heavy metals, the latter of which is poisonous to soil bacteria. Similarly, changes in the structure and activities of soil microbial communities because of mining-related changes in soil physicochemistry could have an impact on key ecosystem processes like soil organic matter turnover, resulting in a decline in overall ecosystem functioning, as well as indirect cascading effects on metal mobility [9].

Certain bacterial features have led to the consideration of microorganisms for bioremediation [13]. Microorganisms are everywhere; they are tiny and multiply quickly, and in vast numbers when exposed to contaminated environments, which makes them viable candidates for bioremediation [14]. They grow tolerant of contaminants and display outstanding degrees of capability to turn pollutants into a source of energy and raw material when exposed to them on a regular basis [15]. They have the ability to evolve genetically to break down pollutants. Tayang and Songachan [13] further maintained that these characteristics could be used to make microbes a perfect choice for a low-cost, environmentally friendly solution. Understanding the physicochemical properties of the soil/substrate and the microorganisms found near mining areas could help influence remediation techniques targeted at lowering heavy metal concentrations or bioavailability.

## Methods

### Sampling area

The local mining area where soil samples were collected was located in Zamfara State, north-west Nigeria, a Sudan savannah zone. The area is characterized by two climatic seasons; dry (November–April) and rainy (May–October). It has a mono-modal rainfall pattern with an annual range from 750 to 1000 mm. The sampling location (Bagega District) from where samples were collected was famous for the presence of illegal mining sites for gold and other precious metals by the local population.

### Sample collection

**Fig. 1:**
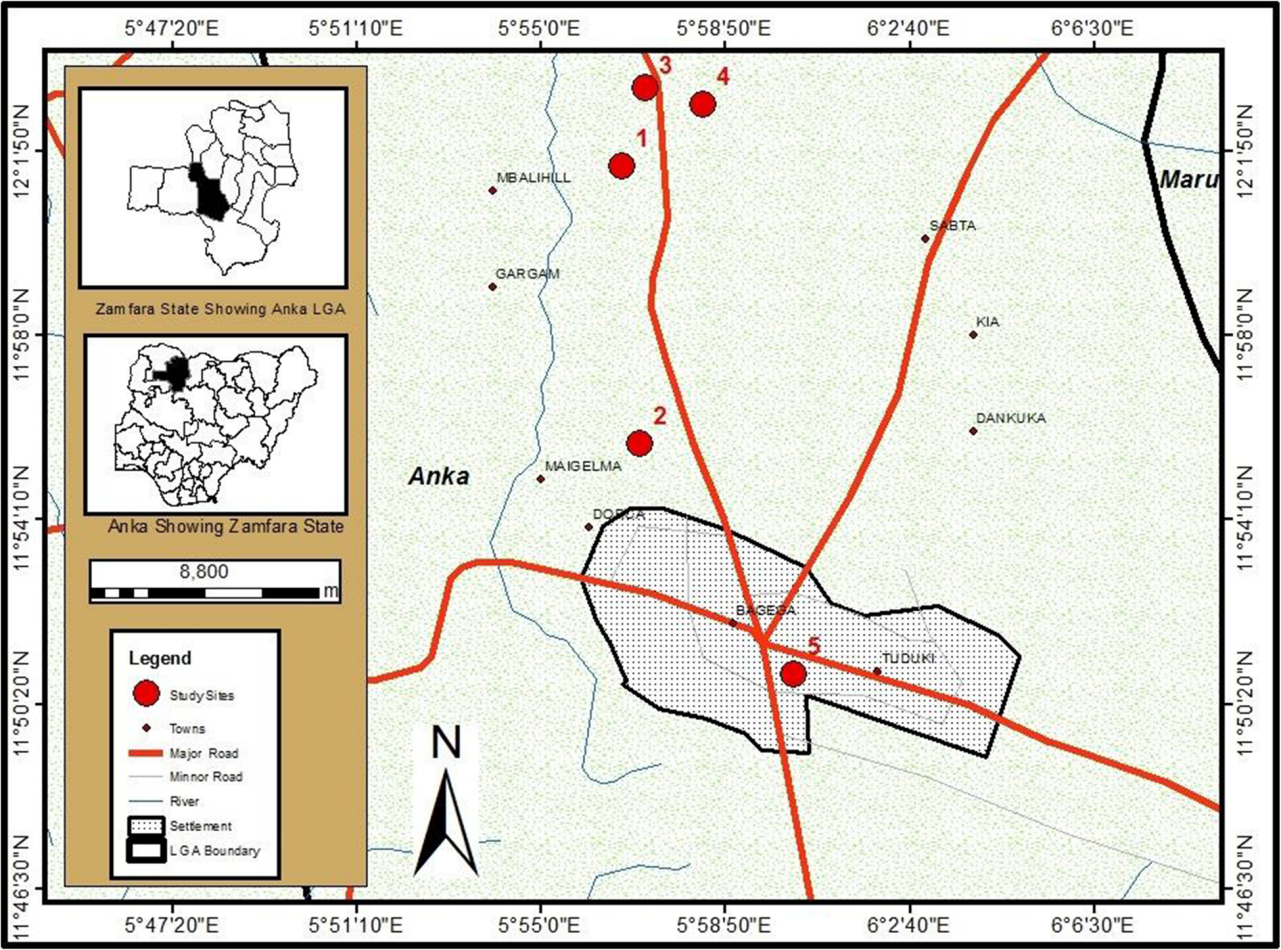
Map and location of sampling sites

**Fig. 2:**
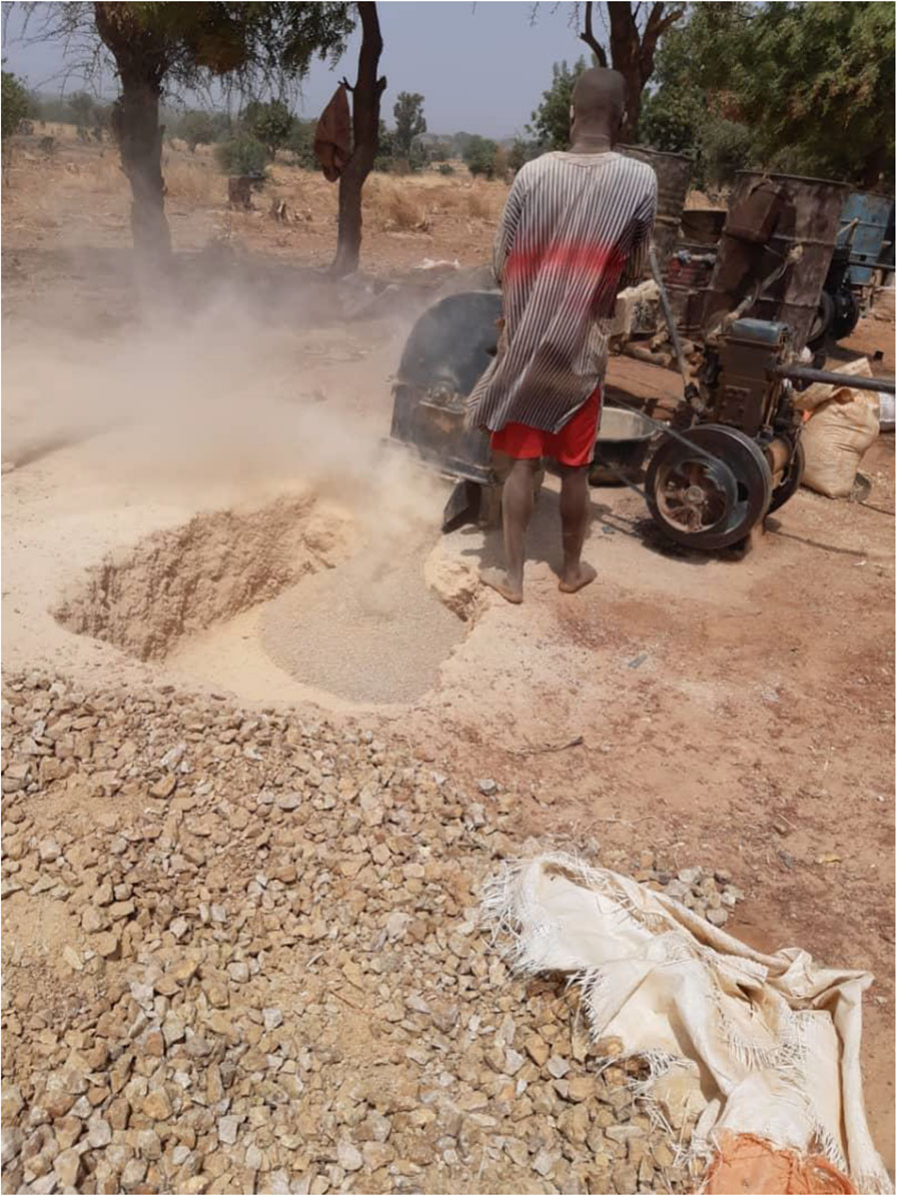
Site 1 of the local mining area

**Fig. 3:**
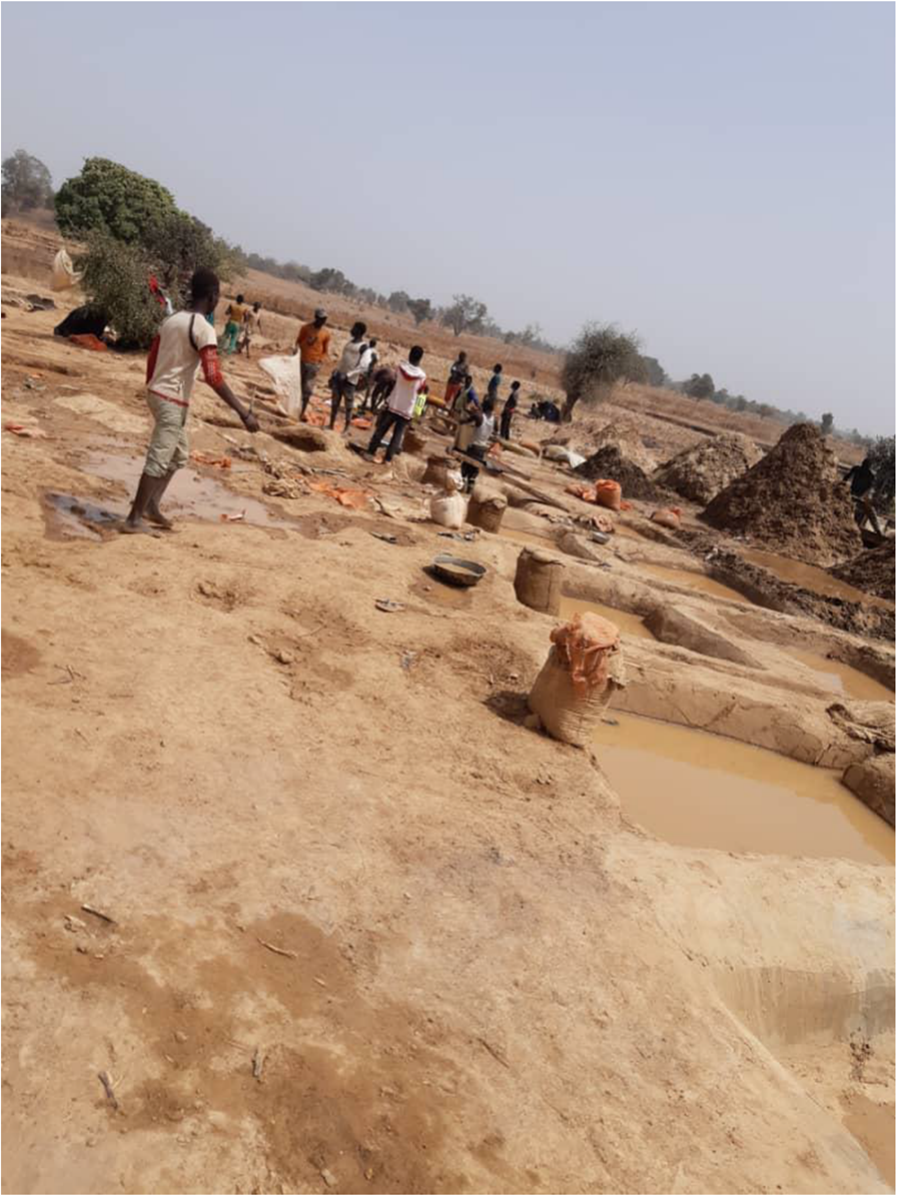
Site 2 of the local mining area

**Fig. 4:**
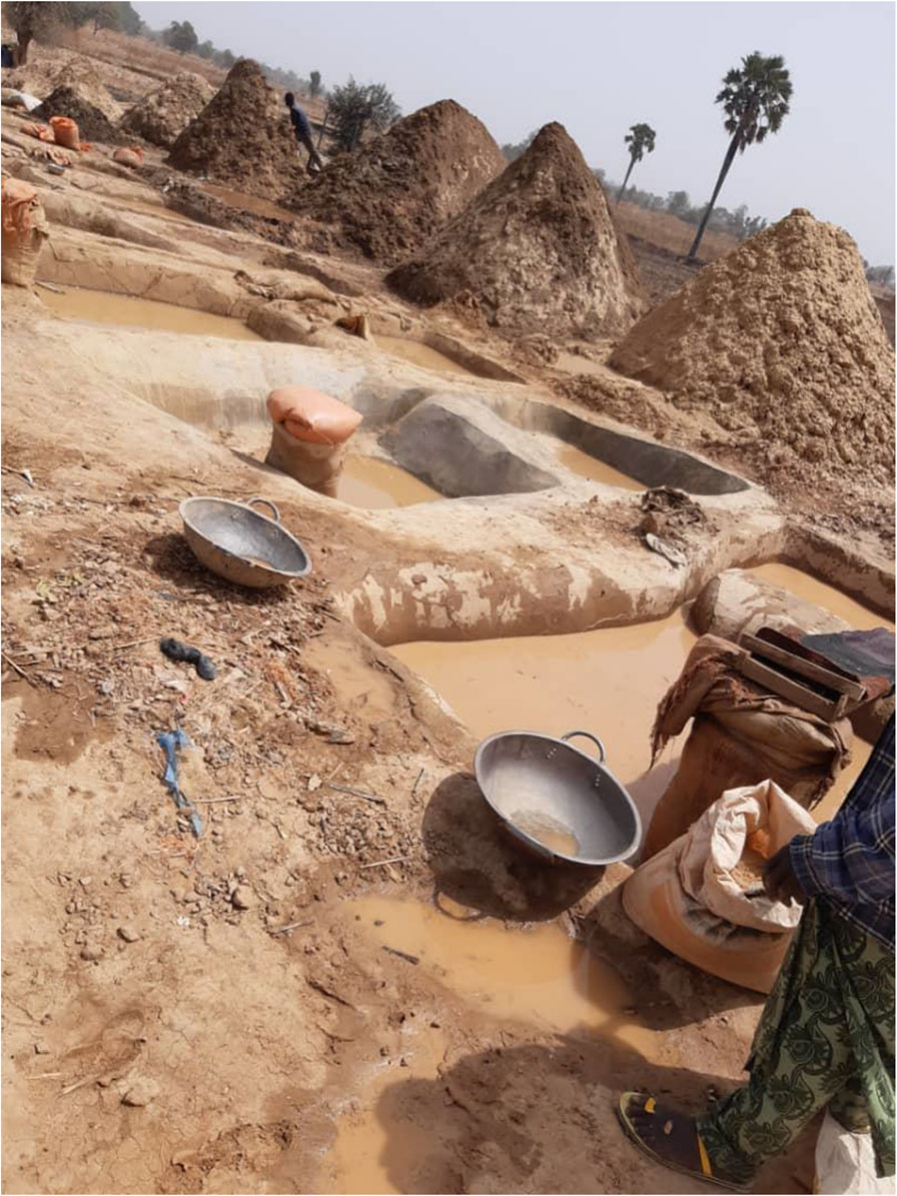
Site 3 of the local mining area

**Fig. 5:**
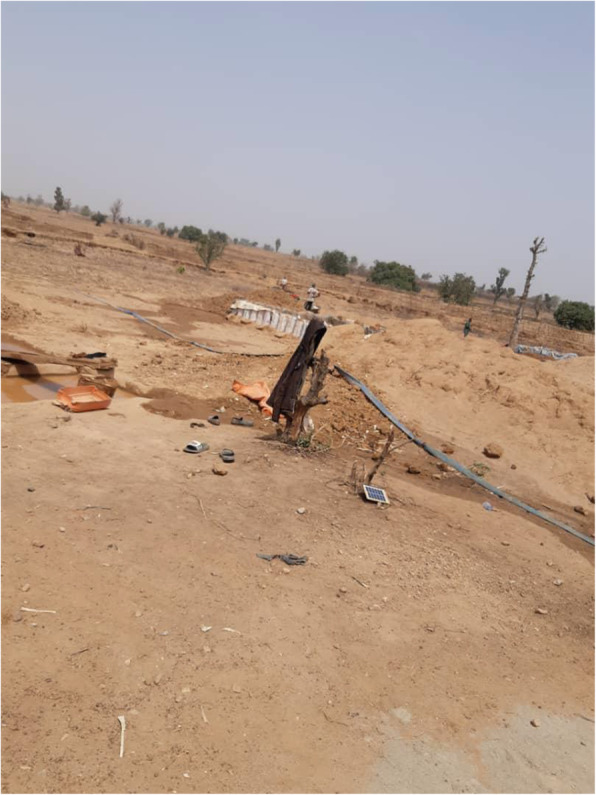
Site 4 of the local mining area

Soil samples were collected from mining sites identified as 1 (Latitude: 12.051 N; Longitude: 5.956 E), 2 (Latitude: 11.992 N; Longitude: 5.959 E), 3 (Latitude: 12.351 N; Longitude: 5.572 E), and 4 (Latitude: 12.352 N; Longitude: 5.581 E) in Bagega District of Anka Local Government Area of Zamfara State. Each composite sample contained bulk soil cores from the surface stratum (0–10-cm depth) taken from sampling points located around the actual mining point (Fig. 1). At each sampling location (shown in Figs. 2, 3, 4, and 5), samples from systematic randomly identified plots, as described by Arotupin et al. [16] and observing USDA [17] protocol of sample collection from mining site soil, were taken and mixed together to form a composite sample. This is to account for the spatial variation that may occur within the soil environment. All analyses carried out in this research are in triplicates.

### Determination of physical and chemical properties of soil

In this study, the temperature [18], pH [19] soil particle size [20], electrical conductivity [19], cation exchange capacity [21], moisture content [22], organic carbon content [19], nitrogen content and stability index, and heavy metals [22] were determined of the soil.

### Enumeration of bacterial loads in soil samples

A stock solution was prepared for serial dilution by dispensing 1 g of soil into 100 ml of distilled water and shaken thoroughly and transferring 1 ml into a test tube containing 9 ml of sterile distilled water and subsequently making a serial dilution of up to 10^5^. Using the spread plate method, 0.1 ml of the suspension from the dilution of 10^3^, 10^4^, and 10^5^ was plated on a prepared nutrient agar (NA) and incubated at 30 °C for 24 h. The results were determined by multiplying the number of counts with the dilution used and expressed as colony-forming units per gram (cfu/g) of soil. Morphological identification used Gram staining and spore staining and relevant biochemical characterizations that include the catalase test, oxidase test, starch hydrolysis test, nitrate reduction test, triple sugar iron test, urease production test, methyl red reaction test, Voges–Proskauer test, indole production test, citrate utilization test, and motility test [23].

### Genomic DNA extraction and PCR amplification

The 16S rRNA gene sequence was amplified by PCR using the universal bacterial primers 27F (5′-AGAGTTTGATCMTGGCTCAG-3′) and 685R (5′-TCTACGCATTTCACCGCTAC-3′). Purified PCR products were sequenced (GATC-Biotech, Konstanz, Germany) and analyzed using BLASTn search [24]. The phylogenetic analysis was conducted for the heavy metal–tolerant bacteria using the method of Feris et al. [25].

### Screening of heavy metal–tolerant isolates and determination of minimum inhibitory concentration

For the determination of metal-tolerant bacteria, the agar plate method was employed for the experiment as described by Shmidt and Schlegel [26] and adopted by Sevgi et al. [27], Oves et al. [28], and Afzal [29]. Varying concentrations (0 (control), 1, 2, 4, 8, 16, 32, and 64 ppm) of chromium chloride (CrCl_3_), lead acetate (Pb(C_2_H_3_O_2_)_2_ ) (1.6 g), cadmium (II) chloride (CdCl_2_) (1.63 g), and copper (II) sulfate (CuSO_4_) (3.9 g) were prepared and incorporated into the nutrient broth medium. Prepared inoculum (0.1 ml) of the isolate standardized by the McFarland standard was spread plated into each of the varying heavy metal supplemented media and incubated in an orbital shaker at 30 °C for 48 h. Heavy metal–tolerant bacterial colonies were isolated by repeated plating; colonies obtained (5%) were recorded compared to control plates (100%). Furthermore, the lowest concentration of a heavy metal that inhibits the growth of the isolate was considered the minimum inhibitory concentration. Data generated were analyzed using descriptive and inferential statistics.

## Results

### Physical and chemical properties of the soil sample

In this study, the physical and chemical characteristics of the soil from the sampling sites determined show the pH values to range from 7.1 to 8.2. The highest pH recorded (8.2 ± 0.14) was obtained from the soil of site 1. Statistical analysis of the data using analysis of variance showed significant differences (*P* ≤ 0.05) between the values existed. The mean temperature of the soil was 38 °C. There was no statistical significance in the values recorded between the soils (*P* ≤ 0.05) (Table 4). The mean range of percentage organic carbon was between 0.18 and 1.12. The highest recorded value was 1.12 ± 0.005% at site 1. Statistical analysis showed a significant difference (*P* ≤ 0.05) between the soils under study (Table 1). Results obtained for total nitrogen indicate a mean value ranging from 0.1 to 0.28%. The highest recorded value of 0.28 ± 0.08% was obtained from site 2 of the sampling location (Table 1). Similarly, statistical analysis revealed a significant difference (*P* ≤ 0.05) existed among the soils. The carbon –nitrogen ratio ranged from 1.8 to 4.8 in the soils under study; this indicated a little free carbon available for microbial use. The cation exchange capacity (CEC) of the soils indicated the highest 3.57 ± 0.26 cmol/kg was determined at site 2. Statistical analysis showed a significant difference (*P* ≤ 0.05) in the CEC value between the soils sampled. Electrical conductivity (EC) of the soil samples studied ranged from 0.15 ± 0.04ds/m to 0.32 ± 0.02ds/m. The highest obtained value was from site 3 of the sampling location, while the lowest EC value recorded was from site 2 (Table 1). Statistical analysis revealed there is a significant difference (*P* ≤ 0.05) between the sampling locations. A noticeable amount of moisture in the soil ranged from 0.14 to 0.82% with the highest value recorded at site 1 (Table 1). The texture of the soil from the sampling location was predominantly of the sandy loam class as determined using soil triangle. The heavy metal with the highest mean concentration in the soils was lead (Pb) with 59.01 ppm obtained at site 3. The lowest concentration was that of cadmium (1.15 ppm) recorded from site 1. Statistical analysis revealed a significant difference (*P* ≤ 0.05) between the soils samples studied (as shown in Table 2).

**Table 1:** Physical and chemical properties of soil obtained from different mining sites of Zamfara State

Parameter	Sampling site				
	Site 1	Site 2	Site 3	Site 4	Site 5 (cntrl)
pH	8.20 ± 0.10^a^	7.17 ± 0.12^b^	7.25 ± 0.15^b^	7.70 ± 0.10^c^	6.20 ± 0.20^d^
Temperature (°C)	40.3 ± 1.6	38.0 ± 0.8	39.6 ± 0.4	39.6 ± 0.4	41 ± 0.8
Moisture (%)	0.82 ± 0.0002^a^	0.63 ± 0.02^a^	0.23 ± 0.006^b^	0.14 ± 0.01^b^	0.57 ± 0.02^c^
Organic Carbon (%)	1.12 ± 0.005^a^	1.01 ± 0.04^a^	0.23 ± 0.01^b^	0.18 ± 0.007^b^	5.92 ± 0.02^c^
Nitrogen (%)	0.23 ± 0.006^a^	0.28 ± 0.008^a^	0.13 ± 0.06^b^	0.1 ± 0.01^b^	0.67 ± 0.02^c^
C/N ratio	4.8	3.6	1.7	1.8	8.8
CEC(cmol/kg)	2.71 ± 0.06^a,b^	3.57 ± 0.26^b^	2.05 ± 0.06^a^	1.52 ± 0.23^c^	2.23 ± 0.33^a^
EC (ds/m)	0.29 ± 0.006^a,b^	0.153 ± 0.04^a^	0.32 ± 0.02^b^	0.26 ± 0.02^a,b^	0.42 ± 0.01^b^
Sand	58	52	62	60	70
Silt	33	27	23	22	30
Clay	9	21	15	14	15
Textural class	SL	SLC	SL	SL	SL
Stability index	3.39	2.68	1.17	1.48	8.26

Key: *cntrl* control, *S* sandy, *L* loam, *C* clay. Note: mean ± standard deviation

^a,b,c,d^Values with common superscripts in the same row do not differ (*p*≤0.05)

**Table 2:** Heavy metal content of soil obtained from soil of local mining sites of Zamfara State, Nigeria

Metal (ppm)	Sampling site					
	Site 1	Site 2	Site 3	Site 4	Site 5 (Control)	MPL in Soil (UNEP, 2013)
Chromium	1.97 ± 0.002^a^	9.85 ± 0.004^b^	5.87 ± 0.23^b,c^	20.95 ± 1.80^d^	0.75 ± 0.000^a^	100
Lead	4.75 ± 0.32^a,b^	2.25 ± 0.002^b^	59.01 ± 3.23^c^	7.02 ± 0.044^a^	0.50 ± 0.002^d^	60
Cadmium	1.15 ± 0.002^a^	1.64 ± 0.004^a^	4.71 ± 0.54^b^	1.20 ± 0.004^a^	1.10 ± 0.01^a^	1.0
Copper	1.42 ± 0.003^a^	1.21 ± 0.001^a^	21.2 ± 2.76^b^	33.0 ± 3.40^c^	1.10 ± 0.022^a^	100

Key: *MPL* maximum permissible limit, *ppm* parts per million. Note: mean ± standard deviation

^a,b,c,d^Values with common  superscripts in the same row do not differ (*p* ≤ 0.05)

### Enumeration and frequency of occurrence of bacteria isolated from soil samples

The enumeration of viable aerobic bacteria shows the highest mean count of 4.5 × 10^6^ cfu/g observed at site 2. Similarly, the lowest bacterial counts recorded (3.3 × 10^4^ cfu/g) were at site 4 (as shown in Table 3). Statistical analysis revealed a significant difference (*P* ≤ 0.05) between the sampled soils of the mining locations. Furthermore, 50% of the isolates identified were Gram negative with the majority being microscopically rod shaped (as shown in Table 4).

**Table 3:** Count of aerobic heterotrophic bacteria from soil of sampling sites

Soil identity	Mean count (cfu/g)
1	2.3 × 10^6^ ± 1.2^b^
2	4.5 × 10^6^ ± 1.7^b^
3	4.0 × 10^4^ ± 0.8^a^
4	3.3 × 10^4^ ± 1.3^a^
5 (control soil)	3.1 × 10^7^ ± 0.6^c^

^a,b,c^Values with common superscript in the same column do not differ (*p*≤0.05)

**Table 4:** Morphological and biochemical characteristics of bacterial isolates from soil of mining areas in Zamfara State

Code	Shape	Spo	Gra	Cat	Lac	Suc	Glu	Cit	Mot	Ind	Ure	MR	VP	Nit.	H_2_S	Gas	Oxi	Sta	Organism
AA	Rod	+	+		−	−	+		+	+	−	+	−	+	−	−	+	−	*Bacillus* sp.
AA1	Rod	−	−	+	−	−	+	+	+	+	−	+	+	+	−	−	+	+	*Pseudomonas* sp.
BA	Rod	−	−	+	+	+	+	+	−	−	−	−	+	+	−	+	−	−	*Aeromonas* sp.
C1	Cocci	−	+	+	+	+	+	+	+	−	−	−	+	+	−	+	−	−	*Streptococcus* sp.
DA	Rod	−	−	+	−	−	−	+	+	−	−	+	−	−	−	−	+	+	*Alcaligenes* sp.
AB	Rod	−	−	+	−	−	−	+	−	+	+	−	−	+	−	−	+	−	*Leptothrix* sp.
BB	Cocci	−	+	+	+	+	−	+	−	+	+	−	+	+	−	+	−	−	*Micrococcus* sp.
DA2	Cocci	+	+		+	+	+		+	−	−	−	−	−	−	−	−	+	*Enterococcus* sp.

Key: *Spo* spore, *Gra* Gram reaction, *Cat* catalase; *Suc* sucrose, *Glu* glusose; *Cit* citrate, *Mot* motility, *Ind* indole; *Ure* urease, *MR* methyl red, *VP* Voges–Proskauer, *Nit* nitrate reduction, *H*_*2*_*S* hydrogen sulfide, *Oxi* oxidase, *Sta* starch hydrolysis

### Bacteriological characteristics of soil samples

**Fig. 6:**
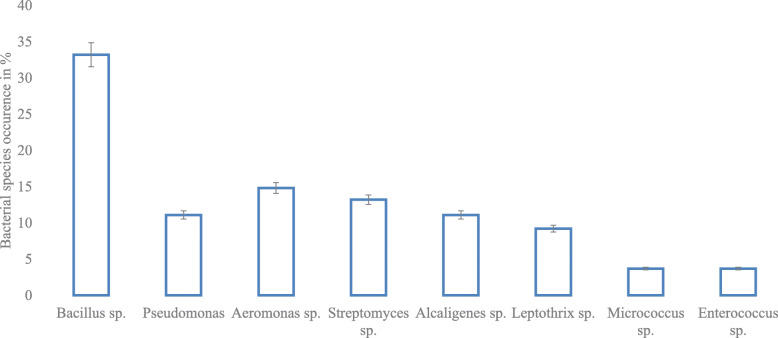
Bacterial species percentage occurrence from the soil of local mining areas

A total of thirty (35) cultivable bacterial isolates from the sampling sites were obtained, excluding the control site. At 33% frequency of occurrence, *Bacillus* sp. was the predominant isolate identified with *Enterococcus* sp. and *Micrococcus* sp. being the lowest at 3.7% (as shown in Fig. 6).

### Screening and selection of heavy metal–tolerant bacteria

**Fig. 7:**
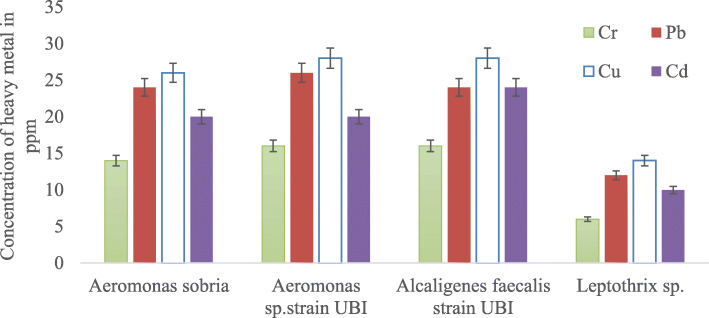
Agarose gel electrophoresis for PCR product of 16SrDNA showing the four bacterial isolates labelled A (*Alcaligenes faecalis* strain UBI), B (*Aeromonas sobria*, C (*Aeromonas* sp strain UBI), and D (*Leptothrix ginsengisoli*)

Screening for heavy metal tolerance performed presented in Table 5 was for the respective isolates identified. The overall heavy metal tolerance of the bacteria isolated from soil of the mining area was presented in Table 6. Subsequent determination of the maximum tolerable limit (MTL) conducted on the tolerant bacteria obtained indicated *Alcaligenes faecalis* strain UBI and *Aeromonas* sp. strain UBI as the most heavy metal tolerant (as shown in Fig. 7).

**Table 5:** Frequency of occurrence of aerobic heterotrophic bacteria from soil of the mining sites

Organism	Site 1	Site 2	Site 3	Site 4	Site 5 (Control)	No. (%)
*Bacillus* sp.	4 (7.04)	3 (5.5)	1 (1.85)	2 (3.70)	8 (14.8)	**18 (33.2)**
*Pseudomonas* sp.	2 (3.70)	3 (5.5)	0 (0.0)	0 (0.0)	1 (1.85)	**6 (11.1)**
*Aeromonas* sp.	2 (3.70)	3 (5.5)	1 (1.85)	0 (0.0)	2 (3.70)	**8 (14.8)**
*Streptococcus* sp.	1 (1.85)	3 (5.5)	0 (0.0)	0 (0.0)	3 (5.5)	**7 (13.2)**
*Alcaligenes* sp.	2 (3.70)	1 (1.85)	1 (1.85)	1 (1.85)	2 (3.70)	**6 (11.1)**
*Leptothrix* sp.	1 (1.85	2 (3.70)	0 (0.0)	0 (0.0)	23.70)	**5 (9.2)**
*Micrococcus* sp.	1 (1.85)	0 (0.0)	0 (0.0)	0 (0.0)	1 (1.85)	**2 (3.7)**
*Enterococcus* sp.	0 (0.0)	1 (1.85)	0 (0.0)	1 (1.85)	0 (0.0)	**2 (3.7)**

**Table 6:** Heavy metal tolerance of the bacterial isolates obtained from soil of mining areas in Zamfara State

Heavy metal	Conc. (ppm)	*Aeromonas* sp.	*Aeromonas sobria*	*Alcaligenes faecalis*	*Bacillus* spp.	*Enterococcus* sp.	*Leptothrix ginsengisoli*	*Micrococcus* sp.	*Pseudomonas* sp.	*Streptococcus* sp.
Pb	˂ 6.0	+	+	+	+	+	+	+	+	+
	≥ 6.0	+	+	+	−	−	+	−	−	−
Cd	˂ 6.0	+	+	+	+	+	+	+	+	+
	≥ 6.0	+	+	+	−	−	+	−	−	−
Cr	˂ 6.0	+	+	+	+	+	+	+	+	+
	≥ 6.0	+	+	+	−	−	+	−	−	−
Cu	˂ 6.0	+	+	+	+	+	+	+	+	+
	≥ 6.0	+	+	+	−	−	+	−	−	−

Key: + : tolerant, − : non-tolerant

### Molecular identification by 16S rRNA analysis

**Fig. 8:**
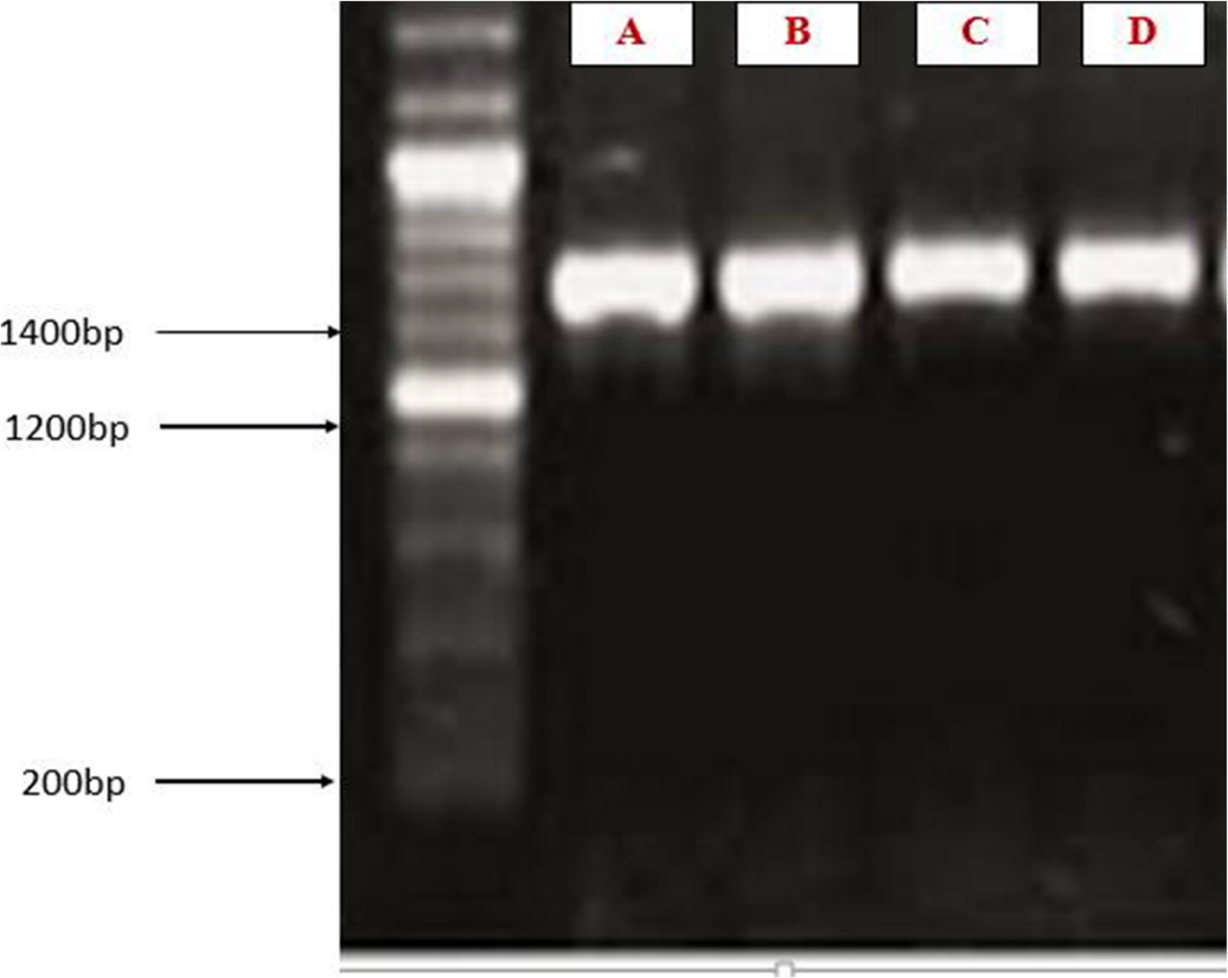
Maximum tolerable level (MTL) of bacteria to different heavy metals

Agarose gel electrophoresis of the bacteria showed 16S rRNA gene amplicons of approximately 1450 bp presenting a separation pattern of PCR-amplified genomic DNA (as shown in Fig. 8). The bacteria belonged to genera *Alcaligenes*, *Aeromonas*, and *Leptothrix* with a variation at the species level. Their query cover and percentage similarity ranged between 60 to 99% and 78 to 99.9% respectively as shown in Table 7. Sequences obtained were submitted to the NCBI data bank and were assigned accession numbers.

**Table 7:** Hit similarity of sequences of the isolates from GenBank of NCBI

Bacteria	Percentage query cover	Percentage identity	Sequence length	Accession no.
*Alcaligenes faecalis* strain UBI	99	99.89	889	MT107249
*Aeromonas* sp. strain UBI	95	84.92	1132	MT126242
*Aeromonas sobria*	95	95.17	654	LC194875.1
*Leptothrix ginsengisoli*	60	78.19	947	EU867315.1

### Phylogenetic analysis of 16S rRNA gene sequence

**Fig. 9:**
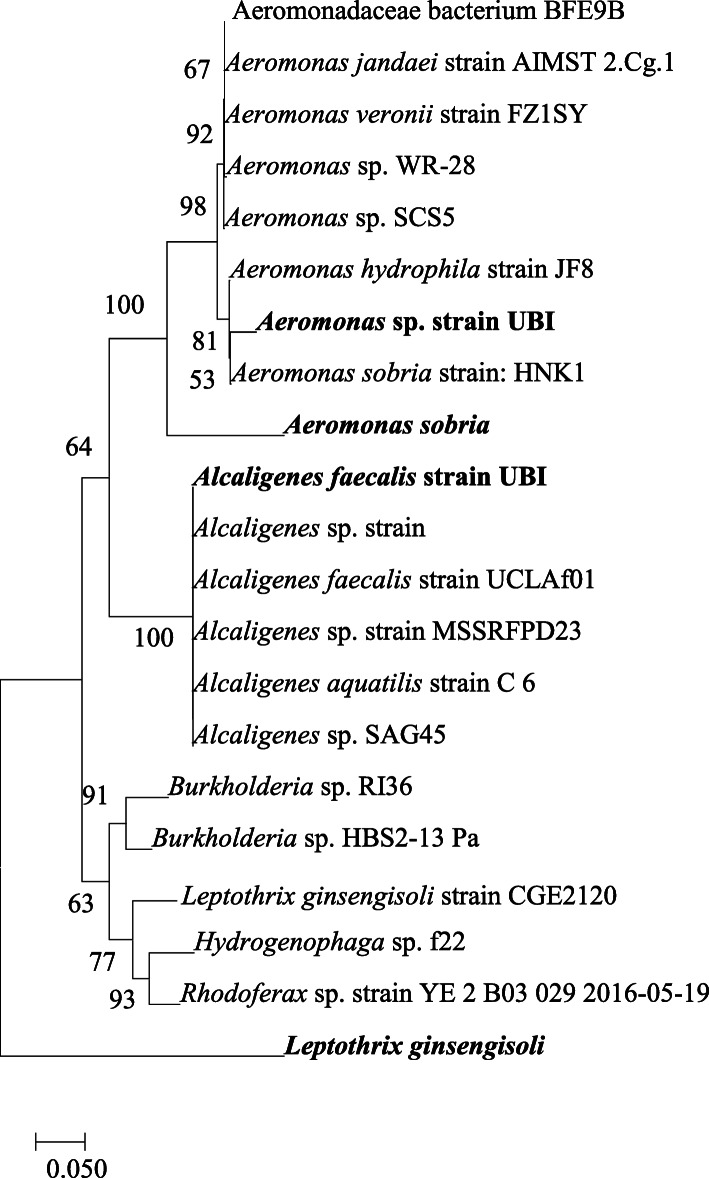
Phylogenetic tree based on 16S rRNA sequence using the neighbor-joining method (bootsrap values were ran at 1000 replications) (isolates in bold are from this study)

The evolutionary relationship is indicated in the phylogenetic analysis as shown in Fig. 9. Further phylogenetic features in the bacteria showed a close relationship in the cluster of *Aeromonas* sp. with the other Aeromonadacea family. A significant relationship noticed was that between *Alcaligenes faecalis* strain UBI and other *Alcaligenes* spp. in the same clade.

## Discussion

In this study, results of the physicochemical analysis of the soil samples showed a mean pH value to range from 7.1 to 8.2. Although both natural and anthropogenic activities can contribute to the variation in pH of soils, Akinnifesi et al. [30] have reported that African soils are slightly acidic, accumulation of other ionic compounds have contributed to the slightly alkaline nature of these soils in this study. This study corroborated with that of Adewole and Adesina [7] and Edema et al. [31] who reported similar findings on physicochemical properties in mining soil. Soil moisture ranged from 0.14 to 0.82, which was low. This result was indicative of the extensive mining activity in the soil, which resulted in a decrease in groundwater and vegetative cover. This finding agreed with the findings of Wang et al. [32] who reported variations in the soil moisture of mining soil. The highest values of soil organic carbon and nitrogen obtained in this study were 1.12% and 0.28%, respectively. Destruction of soil vegetation might be responsible for this impact as studies by Salami et al. [33] reported similar findings on the adverse effects of mining on the soil organic carbon and nitrogen. Soil carbon is an important soil parameter as it improves soil physical and chemical properties and overall soil quality. Soil carbon exists in various forms that are functionally different and have contrasting residence times as reported by Prematuri et al. [34]. The low C:N ratio is reflective of the poor carbon content obtained in the soil, which is obvious in mining soil as the degradation of organic matter increases with regular mining activity. Removal of the topsoil from mining sites and subsequent replacement and mixing with underlying soil considerably reduce the concentration of soil organic carbon/nitrogen ratio as depicted in this study showing the highest approximate value of 5:1 obtained in site 1 which is lower than the average obtainable in natural soils as reported by Gilewska et al. [35]. The ability of soil to exchange cations measured through cation exchange capacity and its electrical conductivity indicated low values across the mining area soil sampled. Lower electrical conductivity (< 0.42 ds/m) in this study is connected to the density of negative charges on the surfaces of soil colloids and the relative charges of metal species on the soil surface [36]. Contrary to the findings in this study, Edema et al. [31] reported higher EC values in a similar study of soil from mining sites in south-south Nigeria. Spatial variation could be the factor responsible for these differences as soil properties tend to differ even at close range.

Assessment of heavy metals between the mining locations under study revealed a significant variation in the concentration between sites. The concentration of Pb found at site 3 was 59.01 ± 3.4 ppm with the least concentration recorded at site 2 (Table 2). The value recorded is higher than the global baseline level of Pb (20 ppm) but higher than the maximum permissible limit (85 ppm) for lead in soil. The variation of concentration among the mining sites sampled as recorded in this study suggests anthropogenic influence in spread as the metal might not have entirely originated from a natural pool of the soil. Similarly, differences in activities between the sites may have played an important role in the variation of concentration of these heavy metals in the area. Studies by Alloway [37], Ma et al. [38], and Demkova et al. [5] had reported similar observations on the widespread accumulation of heavy metals from mining areas. In contrast to what was obtained in this study, Abdu and Yusuf [39] reported a Pb concentration of 235 mg/kg from polluted soils in the Anka Local Government Area of Zamfara State, Nigeria. In another study by Udiba et al. [2], Pb levels recorded in soils of mineral-grinding mills and other selected areas of Anka Local Government in Zamfara State, Nigeria ranged between 346.7 mg/kg to 9010 mg/kg. Similarly, Edema et al. [31] reported a lower (13.0 mg/kg) of Pb in mining soil of Edo State in south-south Nigeria. The soil stability index recorded in this study showed a ranged value of between 1.17 and 3.39. Findings from this study indicated low structural stability across all soils sampled. Frequent and unorganized disruption of the soil structure might have contributed to low values obtained as compared to those of the control soil, which suggests a massive threat to soil aggregates in the mining location. These findings agreed with the study of Zhang et al. [24] who reported massive degradation of former mining soils in the Loess Plateau of China.

Bacterial counts recorded from different mining locations indicated sampling site 2 to have the highest mean count of 4.5 × 10^6^ cfu/g with a significant difference (*P* ≤ 0.05) being observed between the sampling locations. Generally, low counts obtained in these soils may likely be a result of anthropogenic activity and deposition of metal wastes as they are likely to affect microbial community composition. The possibility of thriving resistant species that can tolerate heavy metals at higher concentrations cannot be ruled out. In agreement with this finding, a study by Pires [40] reported scarce counts of bacteria from heavy metal contaminated environments. In contrast to the findings obtained in this study, Margesin et al. [41] reported larger counts of cultivable bacterial communities in heavily contaminated soil polluted by heavy metals. Other studies by Emmanuel et al. [42] reported a lower bacterial count (12.3 × 10^3^ cfu/g) in the soil of mining sites in Nigeria.

The heavy metal–tolerant isolates recovered were Gram negative, which studies [42, 43] showed to be more tolerant to heavy metals than Gram positive. This attribute could be due to the interaction between the bacterial cell wall and the metal ions on the surface and the interface of the bacteria. *Alcaligenes faecalis* strain UBI, *Aeromonas* spp., and *Leptothrix ginsengisoli* isolated in this study tolerated a maximum value of 28 ppm for the metals under study. Bacterial tolerance to higher metal concentrations influenced by chelating, sorption, and complexation properties of surface molecules might be responsible for the attribute. These observations were in agreement with the findings of Abo-Amer et al. [44] who reported the isolation of heavy metal–tolerant *Alcaligenes faecalis* from soil contaminated with heavy metals. Possession of metal-tolerant qualities and adaptation to the primary environment has contributed to their persistence in the culture. Several studies have shown some bacteria to have withstood high metal concentrations [28, 45]. Other studies by Yusuf et al. [46, 47] and Oziegbe et al. [48] reported that some bacteria may tolerate as much as a 100-ppm concentration of heavy metals although isolates from this study showed a much lower tolerance capacity. Furthermore, Marzan et al. [49] reported resistance to individual heavy metals observed in this study in a similar study where bacterial isolates withstood up to 1900 μg/ml of Pb metal, which is lower than what was recorded in this study. This variability in tolerance to different heavy metals might not be unconnected to the source of the isolates and their sensitivity to the heavy metals. Similarly, the degree of toxicity of metals to the isolates might have played an important role in the level of tolerance or otherwise as several studies shows [49, 50]. In conformity to the findings recorded in this study, Jebara et al. [51] reported the isolation of highly Pb-tolerant bacteria from Pb-contaminated soil. Findings in this study were similar to that of Neethu et al. [43] where 3000 μg/l of Pb was identified as the range of concentration that inhibited bacterial growth. Similarly, studies by Andriani et al. [52] reported the isolation of different bacterial species such *Bacillus* sp. and *Enterococcus* sp. in their studies of soil samples from coal mines. In agreement with the findings in this research, Jiang et al. [53] reported the isolation of *Bacillus* sp. that are tolerant to heavy metals obtained from soils around mine refineries. Additionally, Jamal et al. [54] reported the isolation of *Bacillus* sp. from coal mines as the predominant species in their study. Corroborating this study, Qiao et al. [55] reported the isolation of *Bacillus* sp. from lead mine soil. In this study, a heavy metal–tolerant *Aeromonas* sp. strain UBI isolated and characterized conform to the findings of Saleem et al. [56] who isolated similar bacteria from lead-contaminated soil in an industrial estate. Other studies that corroborate the findings of this result are that of Velusamy et al. [57] who reported *Bacillus* sp. as highly tolerant of heavy metals in their studies of heavy metal–tolerant bacteria from hydrocarbon-contaminated soil. Similar findings that agreed to what was observed in this study was that of Karcilic et al. [58] who reported the isolation and tolerant *Micrococcus* sp. from their studies of bacteria isolated from contaminated soil.

The minimum inhibition concentration range for Cu observed in this study was between 15 to 28 ppm. The higher tolerance level can be because of copper being a special co-factor for specific microbial enzymes and involved in microbial pathogenesis, which might be the reason why most isolates develop mechanisms to counteract metal toxicity. Findings in this study were similar to that of Neethu et al. [43] where the MIC for copper to the bacteria was 2000 μg/l. Similar studies by Vicentin et al. [59] reported 314 mg/l as the highest concentration tolerated, which is contrary to that observed in this study. The highest tolerable limit of the isolates to cadmium observed in this study was 24 ppm. Cadmium was a naturally occurring heavy metal and a potential bacterial toxicant having an antagonistic effect. Studies by Oaikhena et al. [60] had reported the tolerance of some bacterial isolates to a maximum of 900 μg/l of cadmium. The findings in this research are also similar to those of Smritha and Usha [61] who reported similar values in terms of cadmium tolerance by bacterial isolates. The least tolerable heavy metal as observed in this study was that of chromium (≤ 1700 μg/l). The presence of chromium in different oxidation states in soil, unlike other metals, has influenced its tolerance. It forms water-insoluble compounds in a non-aqueous solution, which invariably makes it impermeable to cell membrane. In contrast to other metals, chromium also forms cationic species in the oxyanion form, making it difficult to be trapped by anionic components of the bacterial envelope [62].

## Conclusion

In conclusion, there exists a variation with significance in chemical properties of the mining soils sampled such as nitrogen content, organic carbon, and electrical conductivity, but with similar physical properties such as temperature and textural class. Similarly, the stability index value of the soils indicated a degradation-threatened soil in the mining locations sampled. The highest concentration of heavy metals recorded was that of Pb (59.01 ppm) in site 3, and the least concentration recorded was for Cd (1.15 ppm) at mining site 1. Findings from this study recorded the highest count of 4.5 × 10^6^ cfu/g from the soil of mining site 2 indicating low bacterial counts in comparison to non-mining control soil (3.1 × 10^7^ ± 0.6 cfu/g). This study is the first to report a heavy metal tolerance capacity of *Alcaligenes faecalis* strain UBI (MT107249) and *Aeromonas* sp. strain UBI (MT126242**)** isolated from the soil of local mining areas.

## Supplementary Information

**Additional file 1.**

## Data Availability

The datasets used and/or analyzed during the current study are available from the corresponding author on reasonable request. *Alcaligenes faecalis* strain UBI (MT107249) and *Aeromonas* sp. strain UBI (MT126242) data belonging to the authors of this work is at NCBI.
