# Supplementary material for: Physicochemical and molecular characterization of heavy metal–tolerant bacteria isolated from soil of mining sites in Nigeria
Source: J Genet Eng Biotechnol. 2021 Oct 11;19:152. doi: 10.1186/s43141-021-00251-x (PMC8505596; doi:10.1186/s43141-021-00251-x)
Supplement: Supplementary file 1 — Additional file 1. [file 43141_2021_251_MOESM1_ESM.docx]

>Alcaligenes faecalis strain UBI **(THIS STUDY)**

TCGAACGGCAGCACGAGAGAGCTTGCTCTCTTGGTGGCGAGTGGCGGACGGGTGAGTAATATATCGGAAC

GTGCCCAGTAGCGGGGGATAACTACTCGAAAGAGTGGCTAATACCGCATACGCCCTACGGGGGAAAGGGG

GGGATCGCAAGACCTCTCACTATTGGAGCGGCCGATATCGGATTAGCTAGTTGGTGGGGTAAAGGCTCAC

CAAGGCAACGATCCGTAGCTGGTTTGAGAGGACGACCAGCCACACTGGGACTGAGACACGGCCCAGACTC

CTACGGGAGGCAGCAGTGGGGAATTTTGGACAATGGGGGAAACCCTGATCCAGCCATCCCGCGTGTATGA

TGAAGGCCTTCGGGTTGTAAAGTACTTTTGGCAGAGAAGAAAAGGCATCCCCTAATACGGGATGCTGCTG

ACGGTATCTGCAGAATAAGCACCGGCTAACTACGTGCCAGCAGCCGCGGTAATACGTAGGGTGCAAGCGT

TAATCGGAATTACTGGGCGTAAAGCGTGTGTAGGCGGTTCGGAAAGAAAGATGTGAAATCCCAGGGCTCA

ACCTTGGAACTGCATTTTTAACTGCCGAGCTAGAGTATGTCAGAGGGGGGTAGAATTCCACGTGTAGCAG

TGAAATGCGTAGATATGTGGAGGAATACCGATGGCGAAGGCAGCCCCCTGGGATAATACTGACGCTCAGA

CACGAAAGCGTGGGGAGCAAACAGGATTAGATACCCTGGTAGTCCACGCCCTAAACGATGTCAACTAGCT

GTTGGGGCCGTTAGGCCTTAGTAGCGCAGCTAACGCGTGAAGTTGACCGCCTGGGGAGTACGGTCGCAAG

ATTAAAACTCAAAGGAATTGACGGGGACCCGCACAACCGGTGGATGATG

>Aeromonas sp.strain UBI **(THIS STUDY)**

TGCAGTCGAGCGGCAGCGGGAAAGTAGCTTGCTACTTTTGCCGGCGAGCGGCGGACGGGTGAGTAATGCC

TGGGAAATTGCGGGATCAAGGGGGATAACAGTTGGAAACAACTGCTAATACCGCATACGCCCTACGGGGG

AAAGCAGGGGACCTTCCGGCCTTGCGCGATTGTATATGCCCAGGTGGGATTAGCTAGTTGGTGAGGTAAT

GGCTCACCAAGGCGACGATCCCTAGCTGGTCTGAGAGGATGATCAGCCACACTGGAACTGACACACGGTC

CACACTCCTACGGGAGGCAGCAGTGGGGAATATTGCACAATGGGGGAAACCCTGATGCAGCCATGCCGCG

TGTGTGAATAAGGCCTTCGGGTTGTAAAGCACTTTCAGCGAGGAGGAAAGGTCAGTACCTAATATCTGCT

GACTGTGACGTTACTCGCAGAAGAAGCACCGGCTAACTCCGTGCCAGCAGCCGCGGTAATACGGAGGGTG

CAAGCGTTAATCGGAATTACTGGGCGTAAAGCGCACACAGGCGGTTGGATAAGTTAGATGTGAAACCCCC

TGGCTCAACCTGGGAATTGCATTTAAAACTGTCCAGCTAGAGTCTTGTAGAAGGGGGTAGAATTCCACGT

GTAGCAGTGATATGCATAGAAATCTGGAGGAATACCGGTGGCCAAGGAGGCCCCCTGCACGCACACTGAC

GATCACGTGCGAAAGAAGTGGAGCAAAGAGGGATCATATACCCTGGGATGCCCCACCAAAACCGATGGCT

ATTGGACGCAAGGTGGTTTTACCCCCCTCTCCTAAAAAACACAGCAGGGTATTCCAAGGGGAGTCCGAGC

TGACCCAGGGGTTTTTACAATTATAATTAGACCGGCCGCCCGGGCGGGGGGTTTAAGCTATATTATATAA

TAATAACCACCCCCCCCCACCTTTAATCCCGCTCTCTCGTGACAGAAGACGCGGCGGTTTTTCTGGAGCA

GCACCGCCATGAAAAAAATGTATTCACTGGTCTTTCCCCCCCCAAGAAGGAAGATTTACGACAAAGGCCT

CTCATAACCCCACCTTTGTCTTTCTTTGCGTTTCTTCCCTTAAGAAGAAAACCCCCCGCACGCGCTGCCC

GTATGAAAAAAA

>Aeromonas sobria **(THIS STUDY)**

TGCAGTCGAGCGGCAGCGGGGCATCCCTTGCTATTTTTGCCGGCGAGGGGCGAACGGGTGAGTAATGCCT

GCCTGCGTGCCCGGTAGAGGGGGATAACTAGTGGAAACATTTGCTAATACCGCATACGCCCTACGGGGGG

GAGTGGGGGACCTTCGGCCTCATGCTATTGGATGAGCCCATGTCGGATTAGCTATTTGGTGGGGTAACGG

CTCACCAAGGCGACGATCCCTAGCTGGTCTGACACGATGATCAGCCACACTGCCCACTGACACACGGCCC

AGACTCCTACGGGAGGCAGAGTGGGGAATTTTGGACAATGGGGGAAACCCTCATGCAGCGATGCCGCCTG

TGTGTTAAGGCCTTCGGGTTGTAAAGCACTTTCGGCGAGGAGGATTGTATCTGGCTAATACCTGGTGGCT

ATGACGTTACCCGCAGAATAATCACCGGCTAACTACGTGCCAGCATCCGCGGTAATACGGAGGGTGCATT

CGTTAATCTGAATTACTGGGCGGCAAGCGTGCGCAGGCGATTGGAATACATAGATGTGAAAATCCCAGGC

TCAACCTGGAAAGTGTATTGATAACTGTCCAGATGGAGAGTGATATAGGGGTGTACAATTCCCACGTCTA

ACATATATATTTTAATAAAGATGG

>Leptothrix ginsengisoli **(THIS STUDY)**

TCGAGCGGTAAAGGTCTTTTGCTGCTTACTTTTGCCGAAGAGGGGCGTATATGCTTAAACGTCTGCCTTC

TTGTGGGAGACTACTCCAAAAAGGTGCGCTAATACCATATAATCCCAAGAGGAGAAAGGGGGAGATCTTG

GGACCTTGCGCTTGGAGATGACCCTGGGTCATTATGTCTTTTTTGGGAGGTAATGGCCTACCGCCGACAC

TCTGTGTCTGGTCTCAGAGGGGATGATCCGCCACTCTGACACAGAGACACCGCCAACACTCCTACGGGGC

GCCGCTGTGGAGAATTGGGGACTATGGGAGGAAGCCTGATCCCCCCATGCCGCCTGTGAGCGGAAGGCCT

TAGGGTTGTCTGCTTTTGTACCGATGAGGAGGCTACCCTCTGTAATTCTCTGCCCTATTGGTATGACACG

AAAATTACCGGCTAACTACTCCCACACCGCCTAATACGTAAGGTGCTAGCGTTTTCTGAATTTTGGGCGT

AAATCGTGCTCCGCGGTTTTGTAAGACAGAATGAAATCCCCTGGCTCCCCTGGGAACTGCCTTTGTGACT

GCTGGCTAGATTACGGTGAGGGAGGTGGAATTCCGTCTGTATCAATGAAATGCGTAGATATGCATAGGAA

CACCGATGGATAACGAAGGCCCCTGTGCCTCTACTGACTCACATGCACGAAAGCGTGCGGAGCAAACAGG

ATTAAATACCCTGGTAGTCCACGCCCTCACACATGTCAACTAAATGTTGGGTGTTGCCTTCTCGTTAAAA

AGCCACATCTAGAACTTACACTACAGGGGAGGACGTTGCAGGGTGAAACTAAAAAAAAAATACGTGAACC

GCCCACGCAACGGAGAAGAGGTTAAATTAAATCAATCCAAAAACAAAACCATATCTGACTTGGCAGTAAA

TTTCCAAACACAGATCTACTTGTACACTCTACTACTC
